# Chronic capsiate supplementation increases fat-free mass and upper body strength but not the inflammatory response to resistance exercise in young untrained men: a randomized, placebo-controlled and double-blind study

**DOI:** 10.1186/s12970-021-00446-0

**Published:** 2021-06-21

**Authors:** Vilton Emanoel Lopes de Moura e Silva, Jason Michael Cholewa, Ralf Jäger, Nelo Eidy Zanchi, Marcelo Conrado de Freitas, Rayane Carvalho de Moura, Esmeralda Maria Lustosa Barros, Barbara Moura Antunes, Erico Chagas Caperuto, Sergio Luiz Galan Ribeiro, Fabio Santos Lira, Marcos Antônio Pereira dos Santos, Fabrício Eduardo Rossi

**Affiliations:** 1 Immunometabolism of Skeletal Muscle and Exercise Research Group, Department of Physical Education, Federal University of Piauí (UFPI), “Ministro Petrônio Portella” Campus, PI 64049-550 Teresina, Brazil; 2 Department of Exercise Physiology, University of Lynchburg, Lynchburg, VA United States; 3 Increnovo LLC, 2138 E Lafayette Pl, 53202 Milwaukee, WI United States; 4 Laboratory of Cellular and Molecular Biology of Skeletal Muscle (LABCEMME), Department of Physical Education, Federal University of Maranhão, São Luís, Brazil; 5 Department of Nutrition, University of Western São Paulo (UNOESTE), Presidente Prudente, Brazil; 6 Department of Biophysics and Physiology, Federal University of Piaui, Campus Minister Petrônio Portela, Ininga, Teresina, Piaui Brazil; 7 Exercise and Immunometabolism Research Group, Department of Physical Education, São Paulo State University (UNESP), Presidente Prudente, SP Brazil; 8 University São Judas Tadeu, São Paulo, SP Brazil; 9 Associate Professor at Graduation Program in Science and Health, Federal University of Piaui (UFPI), Teresina-PI, Brazil

**Keywords:** Pre-workout, Hypertrophy, Performance, Strength training

## Abstract

**Background:**

Acute capsaicinoid and capsinoid supplementation has endurance and resistance exercise benefits; however, if these short-term performance benefits translate into chronic benefits when combined with resistance training is currently unknown. This study investigated changes of chronic Capsiate supplementation on muscular adaptations, inflammatory response and performance in untrained men.

**Methods:**

Twenty untrained men were randomized to ingest 12 mg Capsiate (CAP) or placebo in a parallel, double-blind design. Body composition and performance were measured at pre-training and after 6 weeks of resistance training. An acute resistance exercise session test was performed pre and post-intervention. Blood samples were collected at rest and post-resistance exercise to analyze Tumor necrosis factor- (TNF-), Soluble TNF- receptor (sTNF-r), Interleukin-6 (IL-6) and Interleukin-10 (IL-10).

**Results:**

Exercise and CAP supplementation increased fat-free mass in comparison to baseline by 1.5 kg (*P* < 0.001), however, the majority of the increase (1.0 kg) resulted from an increase in total body water. The CAP change scores for fat-free mass were significantly greater in comparison to the placebo (CAP ∆%= 2.1 ± 1.8 %, PLA ∆%= 0.7 ± 1.3 %, *P* = 0.043) and there was a significant difference between groups in the bench press exercise (*P =* 0.034) with greater upper body strength change score for CAP (∆%= 13.4 ± 9.1 %) compared to placebo (∆%= 5.8 ± 5.2 %), *P* = 0.041. CAP had no effect on lower body strength and no supplementation interactions were observed for all cytokines in response to acute resistance exercise (*P* > 0.05).

**Conclusion:**

Chronic Capsiate supplementation combined with resistance training during short period (6 weeks) increased fat-free mass and upper body strength but not inflammatory response and performance in young untrained men.

**Supplementary Information:**

The online version contains supplementary material available at 10.1186/s12970-021-00446-0.

## Background

Capsaicin is a bioactive phytochemical compound found primarily in chili peppers and other spicy foods, and has been purported to increase metabolism [1], muscular performance [2], relieve pain [3], and improve the immune system function [4]. Capsiate (CAP) is chemical analogs of capsaicin with an ester linkage replacing the amide bond between the vanillyl moiety and the fatty acid chain [5].

In humans, acute CAP supplementation increased the total volume of exercise during an acute resistance exercise session performed to muscular failure and decreased the rate of perceived exertion in a trained population [6]. Similar findings of increased muscle performance were also observed in mice, where chronic capsaicin supplementation increased forelimb grip strength in a dose dependent manner [7]. Although the mechanisms responsible for increased muscle performance are not fully elucidated in skeletal muscle, capsaicin has been shown to activate the transient receptor potential vanilloid-1 (TRPV1), thus increasing the release of calcium (Ca^+^) by the sarcoplasmic reticulum [8]. Given the crucial role that that Ca^+^ ions exert on muscle contraction, such increases may also positively influence force generation [9].

Exercise training has been shown to induce profound changes in muscle physiology. For example, during muscle contractions, IL-6 gene expression is activated by Ca^+^ dependent pathways, resulting in a transient, but potent increased IL-6 secretion. In this regard, Obi et al. [4] demonstrated that capsaicin was able to activate the TRPV1 in myoblasts leading to an increase in interleukin-6 (IL-6) mRNA expression. The importance of increased systemic IL-6 for the immune system relies in its potential anti-inflammatory response. Increased IL-6 secretion by contracting skeletal muscles has been demonstrated to inhibit tumor necrosis factor alpha (TNF-a) and increases the release of IL-10 by immune cells [10]. In fact, in a previous study conducted by Zanchi et al. [11], resistance training was capable to increase the IL-10/TNF-a ratio in muscle samples from rat muscle. However, since muscle samples also contain other cells types, the exact source responsible for such changes were not evaluated.

Since capsaicin/CAP increases muscle IL-6 mRNA gene expression [4], and since exercise training is a potent stimulus for IL-6 secretion [10], it is reasonable to hypothesize that both strategies may present synergistic anti-inflammatory effects. Moreover, since acute capsaicin/CAP increases muscle performance [2] and energy expenditure [1], these effects may summate when supplemented during chronic exercise training, thus promoting additional adaptations. Hypothetically, such benefits will manifest not only in the immune system, but also in the muscle performance and perhaps, in body composition.

Therefore, the main objective of the present study was to test if CAP supplemented before resistance training sessions would be capable to summate and produce a chronic synergistic effect. Specifically, reduced inflammatory response, improved anti-inflammatory response, increased muscle performance and enhanced body composition are hypothesized to occur in a CAP plus resistance training group compared with a placebo plus resistance training mainly in untrained subjects, since they could exhibit greater inflammation than trained subjects using the same load and maybe higher response with intervention. If confirmed, CAP could be utilized in combination with resistance training to potentiate the already known benefits of resistance training, in the supra-cited variables.

## Methods

### Experimental design

**Fig. 1:**
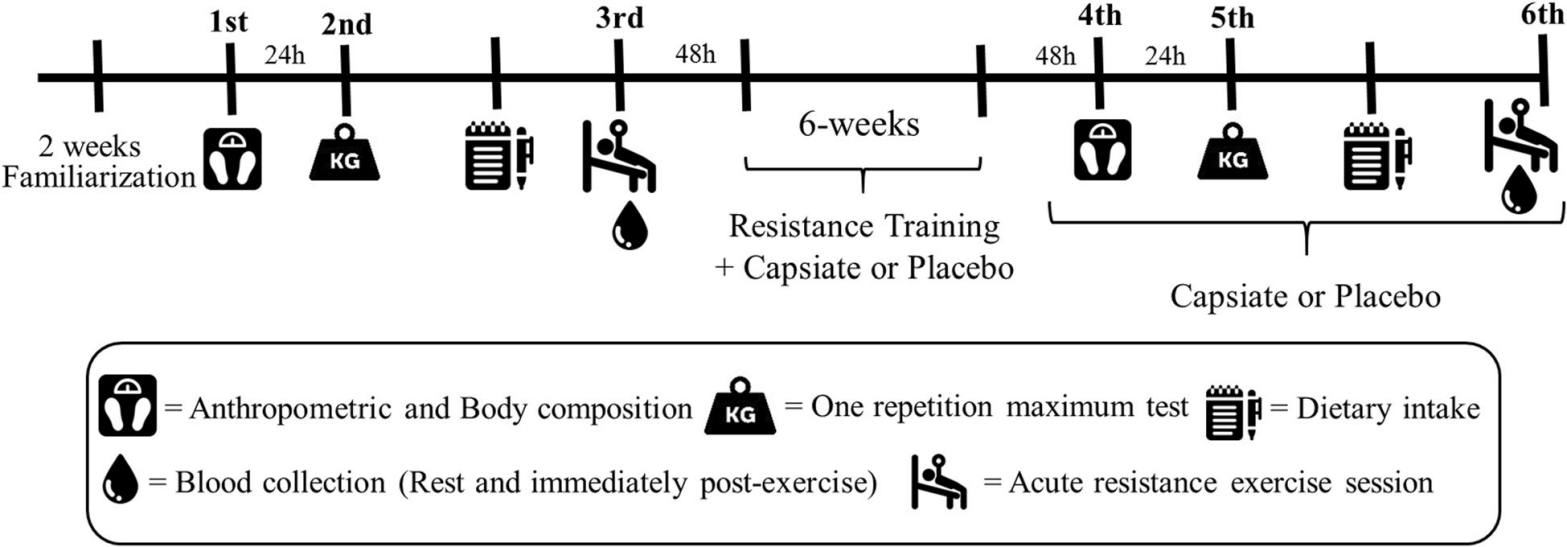
Experimental design

Firstly, the participants completed two weeks of familiarization with the exercise program routine. On the first visit to the laboratory, the participants were assessed for anthropometrics and body composition. On the second visit, 24 h later, the one repetition maximum test (1RM) was performed. Next, participants were pair-matched based on initial fat-free mass, fat mass and strength levels, and then randomly allocated to one of two treatment groups (CAP or Placebo). Food records were distributed to all participants pre and post-intervention to record food intake for three nonconsecutive days (one weekend and two weekdays), where one weekday corresponded to the day before the acute resistance exercise session. The third visit was separated by 72 h and subjects performed the acute resistance exercise session test. Blood samples were collected 90 min post-prandial (rest) and immediately following the acute resistance exercise session test. After 48 h, both groups participated in a 6-week progressive resistance training program, as shown in Supplementary Table 1, combined with placebo or CAP supplementation, and then returned to laboratory at post-training to repeat all assessments (4th to 6th visits). Supplementation continued through the post-training evaluation phase. The experimental design is illustrated in Fig. 1.

### Participants

This randomized, placebo-controlled and double-blind study was carried out according to the 2013 Revision of the Declaration of Helsinki and it was approved by the Institutional Review Board from Federal University of Piaui in February, 2019 (Protocol number: 3.169.545) according the ethical standards [12]. Participants were invited through media and newspaper advertising at campus to participate in the study. The participants contacted the researchers by phone and an appointment was made in order to carry out a more detailed interview.

The inclusion criteria for the present study were: (1) males between 18 and 30 years of age; (2) had not participated in regimented resistance training in the previous 6 months; (3) had not used any ergogenic supplement for at least two years prior to the study; (4) did not smoke or drink alcohol within of testing visits (1st to 3rd visit at baseline and 4th to 6th visit at post-training); (5) no contraindications involving the cardiovascular system, muscles, joints, or bones of the lower and upper limbs that could limit exercise. All participants signed a consent form and were informed about the purpose of the study and the possible risks. During the study, all participants were instructed not to use any other supplement or ergogenic substance and to maintain their current diets. Additional exclusions criteria included missing more than 3 workouts during the intervention.

Out of a total of 58 men who participated in the first screening, 28 met all the inclusion criteria and 22 agreed to participate in the study protocol. This study used a randomized, double-blind design. Participants were pair-matched based on initial fat-free mass, fat mass and strength levels and then randomly allocated to one of two treatment groups: CAP (n = 11) and placebo (n = 11). During the intervention, one participant from placebo group dropped out of the study due to unspecified reasons and there was an outlier in the same group who was excluded from the final analysis due higher fat mass in relation to sample. It was calculated as the ratio Z as the difference between the outlier and the mean divided by the SD (via (https://www.graphpad.com/quickcalcs/grubbs1/) and the outlier comes from a different population if Z is greater than 1.96. Thus, the final sample analyzed was 11 subjects in the CAP group and 9 in the placebo group.

### Procedures

#### Dietary intake assessment and supplementation procedure

Food records were distributed to all participants pre and post-intervention to record food intake for three nonconsecutive days (one weekend and two weekdays), where one weekday corresponded to the day before the acute resistance exercise session. A breakfast (total energy 603 ± 65 kcal, consisting of 25 % protein, 50 % carbohydrates and 25 % fat) was provided to participants before the acute resistance exercise (1 h 30 min). The software Dietbox (version 3.3.0) utilized the database of Brazilian food composition table (TACO) to calculate dietary intake. The volunteers were instructed not to consume chili peppers or other spicy foods and any kind of supplementation during the study, as well as coffee, tea, alcohol and/or stimulant drinks for a period of 12 h prior to assessments.

Each participant randomly consumed 2 capsules of placebo (starch) or CAP with 6 mg Capsiate per capsule (12 mg Capsiate in total), which were identical in appearance to ensure a double-blind design, 45 min before each experimental test and before lunch on non-training days. This time was selected because capsaicin reaches maximum concentrations within 45 min after ingestion [12]. CAP was standardized to contain 50 % Capsiate (Capsicum annuum L.) (Purifarma-Gemini Pharmaceutical Industry Ltda, Anapolis, GO, Brazil). Study products were delivered to each individual subject by a person who was not directly involved in the data collection to ensure blinding.

#### Anthropometric and body composition measurements

Body weight was measured using an electronic scale (Filizola PL 50, Filizzola Ltda., Brazil), with a precision of 0.1 kg. Height was measured on a fixed stadiometer with an accuracy of 0.1 cm and a length of 2.20 m. The total body water (TBW), intracellular (ICW) and extracellular water (ECW), fat-free mas (FFM), fat mass (FM), and percentage of fat mass (FM%) were assessed using a spectral bioelectrical impedance analysis and accompanying software (InBody S10, Gangnam-gu, Seoul, Korea). Based on results of a small pilot study (*n* = 8), the test retest intraclass correlation coefficient (ICC) from our lab were TBW (0.99), ECW (0.99), ICW (0.98), FM (0.97) and FFM (0.99).

#### Blood samples and analyses

Blood samples (20 ml) were collected at pre-training and after 6 weeks of training at rest and immediately post-exercise (within 5 min) during the acute resistance exercise session. The tubes were centrifuged at 3000 rpm for 15 min at 4ºC, and plasma and serum samples were stored at -80ºC until analysis. The Tumor Necrosis Factor-Alpha (TNF-α), soluble TNF-α receptor (sTNF-r), IL-6 and IL-10 serum concentrations were determined by enzyme-linked immunosorbent assay (ELISA) technique using high detection sensitivity kits (R&D System®, Minneapolis, MN, United States) with range between 15.6 and 1000 pg/mL for TNF-α, 7.8–250 pg/mL for sTNF-r, 3.13–300 pg/mL for IL-6 and 7.8–500 pg/mL for IL-10; and intra- and inter-assay variation (%) of 4.2–5.2 and 4.6–7.4 (TNF-α), 3.6-5.0 and 3.7–8.8 (sTNF-r), 1.6–4.2 and 3.3–6.4 (IL-6) and 1.7-5.0 and 5.9–7.5 (IL-10), respectively.

#### Muscular performance tests and resistance training protocol

Prior to all pre-training muscular performance tests, the participants completed two weeks of low-intensity familiarization with the exercises listed in Supplementary Table 1 with 2 sets of 15 repetition in each exercise and 60 s of rest. Subjects completed a warm-up before each test, which consisted of 5 min of walking (~ 6 km.h^− 1^) and a subsequent set of 10 repetitions at approximately 50 % of the 1RM for the first exercise of the specific workout of the day, according to Supplementary Table 1.

Lower and upper body strength was assessed by 1RM testing in the 45º leg press exercise (1RM-leg press) and the bench press (1RM-bench press) exercises, according the National Strength and Conditioning Association [13].

The acute resistance exercise session consisted of 3 sets of 45º leg press followed by 3 sets of bench press until momentary muscular failure with a load corresponding to 70 % of the 1RM and 90 s of rest between sets and exercise, according to Conrado de Freitas et al. [6] Both peak and mean power output were recorded for each repetition using the Tendo™ Power Output Unit (Tendo Sports Machines, Trencin, Slovak Republic) and the peak power for 3 sets (watts) and mean power for 3 sets (watts) were used [14].

The chronic resistance training program consisted of two progressive phases (Supplementary Table 1) and the intensity was controlled by zone of repetition. The sets were executed until concentric muscular failure (i.e. when the participants performed the training with repetitions varying from 10 to 12 RM, they were to execute at least 10 and no more than 12 RM). In the case of the participants executing more repetitions, the load was increased in order to keep within the training zone [15]. Fitness professionals supervised all testing, the exercise routine was conducted face to face (one fitness professional trained two participants), and the total number of repetitions performed was recorded for each set and each exercise, and the total weight lifted was calculated (repetitions × weight lifted × sets). The sum of the total weight lifted for each day was calculated to show total volume per week across the study.

### Statistical analysis

Using a partial eta-square (0.26) for fat-free mass in the present study, and an alpha value of 5 % via GPower 3.1 software, a 99 % power to our sample size was found by analyzing the difference between groups post hoc. For the outcome measures, an Analysis of covariance (ANCOVA) was applied to adjust for pre-intervention and an Independent *t* test was used to verify the difference between relative changes (∆%= post-exercise value minus rest value/rest*100) and confidence interval (CI 95 %) was calculated. For the acute resistance exercise analysis, a mixed between–within subject repeated measures ANOVA was used where the supplementation group (placebo vs. capsaicin) was included as the between-subject factor, exercise effect (rest vs. post-exercise) and time (pre-training vs. after 6-weeks of training) were used as the within-subject factors. The inflammatory data was transformed to log, since the data presented non-parametric distribution, according to the Shapiro-Wilk test and a mixed between–within subject ANOVA was used again. The estimated sphericity was verified according to Mauchly’s W test and the Greenhouse-Geisser correction was used when necessary. Effect sizes for the ANOVA were calculated using partial eta squared (*η*^2^) for time, group and interaction. Data are presented as mean and standard deviation (SD). Statistical significance was set at p < 0.05. The data were analyzed using the Statistical Package for Social Sciences 17.0 (*SPSS* Inc. Chicago. IL.USA). In addition, it is important highlight that statistician was blind to both groups during data analyses.

## Results

The Supplementary Table 2 shows the total kilocalorie, macronutrient and micronutrient intakes averaged for the three days expressed as grams or relative to body mass at pre-training and post-intervention. There were no statistically significant differences between groups for dietary intake (kcal), carbohydrate, proteins, fats and all micronutrients.

### Chronic effect of CAP combined with resistance training

Table 1 presents the comparison between Capsiate and placebo groups for body composition and performance pre and post-intervention.

**Table 1:** Comparison between Capsiate supplementation and placebo groups on the body compositionand performance. Data are presented as mean +/- SD

***Body Composition***	**Pre-intervention**	**After 6 weeks**	***ANCOVA p-value***	***ES***
**Body mass (kg)**				
Capsiate	69.9 ± 11.9	70.8 ± 11.6	0.825	0.08
Placebo	68.3 ± 11.5	69.1 ± 11.2		0.07
**Fat-Free mass (Kg)**				
Capsiate	57.9 ± 7.8	59.2 ± 7.8 ^¥^	0.024	0.20
Placebo	58.7 ± 7.7	59.0 ± 7.2		0.04
**Fat mass (Kg)**				
Capsiate	11.9 ± 4.9	11.6 ± 4.6	0.340	-0.06
Placebo	9.8 ± 5.4	10.6 ± 5.6		0.15
**Body fat (%)**				
Capsiate	16.5 ± 5.2	15.9 ± 4.6	0.333	-0.12
Placebo	13.9 ± 5.7	14.1 ± 5.9		0.03
**Extracellular water (L)**				
Capsiate	15.8 ± 2.2	16.2 ± 2.1	0.477	0.20
Placebo	15.9 ± 2.1	16.1 ± 2.2		0.09
**Intracellular water (L)**				
Capsiate	26.5 ± 3.4	26.9 ± 3.4	0.842	0.12
Placebo	26.7 ± 3.8	27.1 ± 3.6		0.11
**Total body water (L)**				
Capsiate	41.9 ± 5.4	42.9 ± 5.4	0.198	0.20
Placebo	42.5 ± 5.9	43.0 ± 5.7		0.09
***Performance***				
**Muscle strength at 45 leg press (kg)**				
Capsiate	276.4 ± 77.1	380.6 ± 86.5	0.426	1.30
Placebo	292.2 ± 55.9	388.9 ± 56.0		1.70
**Muscle strength at bench press (kg) **				
Capsiate	55.2 ± 16.6	61.7 ± 16.4^¥^	0.034	0.40
Placebo	60.6 ± 14.7	63.9 ± 15.1^¥^		0.22
**Peak power for 3 sets (watts)**				
Capsiate	2793.4 ± 808.3	3753.1 ± 715.9	0.690	1.26
Placebo	2646.6 ± 756.9	3514.4 ± 1073.2		0.95
**Mean peak power for 3 sets (watts)**				
Capsiate	2301.2 ± 636.7	3201.3 ± 551.7	0.819	1.51
Placebo	2003.6 ± 644.7	3177.6± 943.8		1.48

*ES* effect size, *ANCOVA *Analysis of covariance *used* to adjust for *pre-intervention*

*Note*: ¥=Bonferroni’s Post hoc with statistically significant difference compared to Pre

**Fig. 2:**
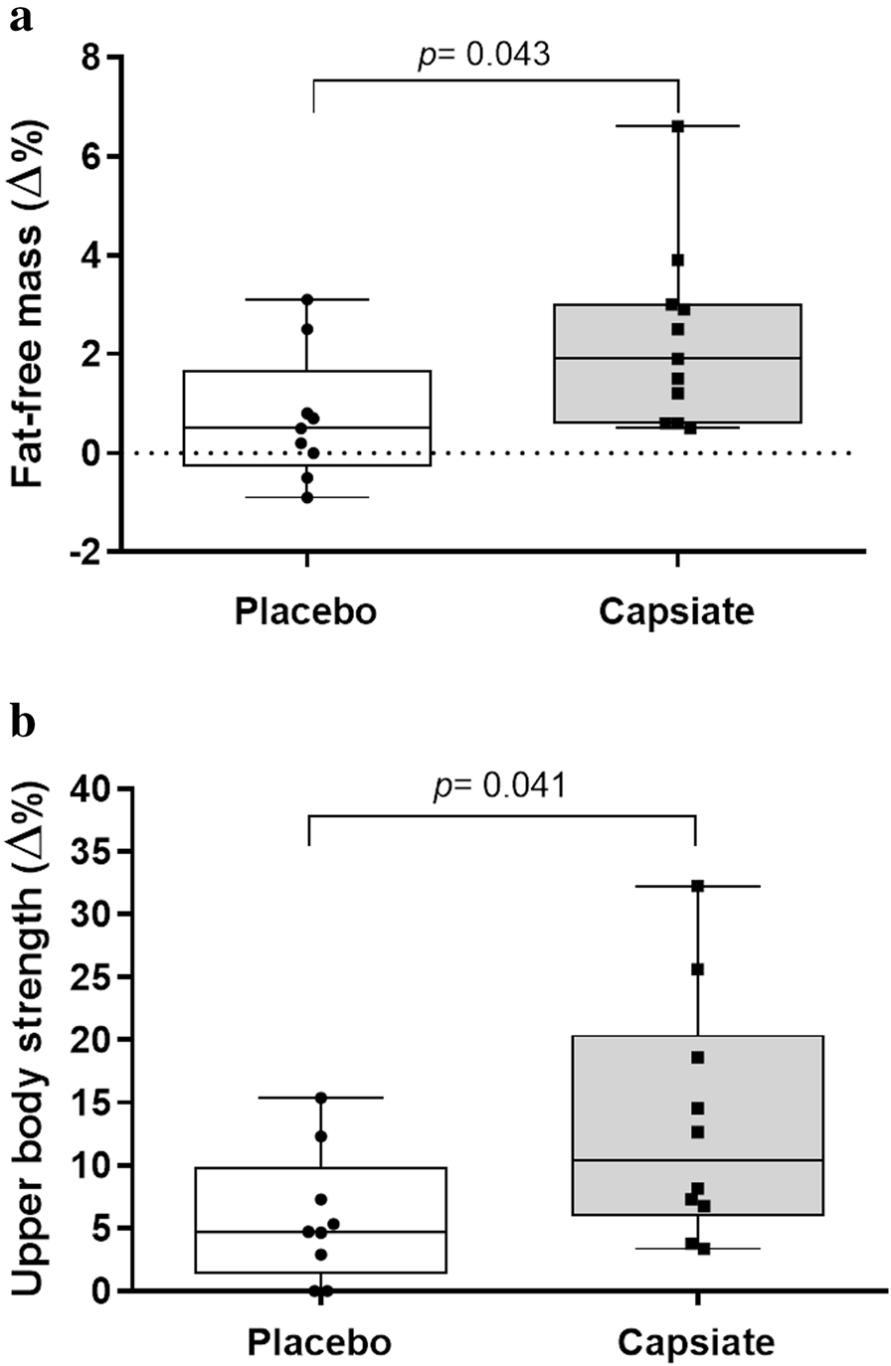
Relative changes and individual response in the fat-free mass (**a**) and upper body strength (**b**) after 6 weeks of intervention in the Capsiate supplementation and placebo groups

For fat-free mass, ANCOVA analysis showed a significant difference between groups (*F* = 6.610, *P* = 0.024, *η*^*2*^ *=* 0.26). When we analyzed the percentage of changes, there was statistically significant differences between group (*t*= -2.181, *P* = 0.043) with a greater increase for CAP (∆%= 2.1 ± 1.8 %, CI 95 %= 1.2 to 3.4) in relation to placebo (∆%= 0.7 ± 1.3 %, CI 95 %= -0.2 to 1.6) (Fig. 2). When we adjusted the FFM effect by the amount of protein consumption, the significant group × time interaction remained (*F* = 5.725, *P* = 0.029, *η*^*2*^ *=* 0.26).

There were no a significant difference between groups for total body mass (*F* = 0.050, *P* = 0.825, *η*^*2*^ *=* 0.03), fat mass (*F* = 0.964, *P* = 0.340, *η*^*2*^ *=* 0.05), and percentage of fat mass (*F* = 0.993, *P* = 0.333, *η*^*2*^ *=* 0.05). There were no significant difference between groups for total body water (*F* = 1.794, *P =* 0.198, *η*^*2*^ *=* 0.09), extracellular (*F* = 0.041, *P =* 0.842, *η*^*2*^ *=* 0.002) and intracellular water (*F* = 0.529, *P =* 0.477, *η*^*2*^ *=* 0.03).

For muscle strength in the bench press, ANCOVA analysis showed significant difference between groups (*F* = 5.283, *P =* 0.034, *η*^*2*^ *=* 0.24). When analyzed the percentage of changes across the intervention, there was statistically significant differences between treatments (*t*= -2.204, *P* = 0.041) with greater upper body strength for CAP (∆%= 13.4 ± 9.1 %) compared to placebo (∆%= 5.8 ± 5.2 %).

There were no significant differences between groups for muscle strength in 45º leg press exercise (*F* = 0.665, *P =* 0.426, *η*^*2*^ *=* 0.04), power output (*F* = 18.789, *P =* 0.001, *η*^*2*^ *=* 0.56) and mean peak power (*F* = 0.054, *P =* 0.819, *η*^*2*^ *=* 0.003) during the acute resistance exercise session.

### Acute resistance exercise session performed at baseline and after 6 weeks of intervention

The Table 2 presents the total number of repetition for the acute resistance exercise session in leg press and bench press.

**Table 2:** Comparison of the total number of repetition for acute resistance exercise at pre and post-intevention

Exercise	Group	Pre-intervention						After 6 weeks				
		**Set-1**	**Set-2**	**Set-3**	**Total**	**TWL**		**Set-1**	**Set-2**	**Set-3**	**Total**	**TWL**
**Leg press (reps)**	**Group1**	20.6 ± 2.9	12.9 ± 2.1 ^a^	10.9 ± 1.8 ^a.b^	44.3 ± 5.7	12820.0 ± 2222.3	**Placebo**	25.4 ± 4.9	16.3 ± 3.5 ^a^	11.2 ± 2.0 ^a.b^	53.0 ± 9.7*	20675.0 ± 5036.7*
	**Group2**	22.4 ± 3.7	12.9 ± 3.5^a^	10.1 ± 2.3 ^a.b^	45.4 ± 7.7	12429.1 ± 4085.7	**CAP**	28.6 ± 2.7	17.6 ± 4.8 ^a^	11.8 ± 1.8 ^a.b^	58.1 ± 8.5*	21962.3 ± 5497.7*
**Bench press (reps)**	**Group1**	14.1 ± 3.4	6.2 ± 1.2 ^a^	2.9 ± 0.6 ^a.b^	23.2 ± 4.0	1395.9 ± 368.8	**Placebo**	16.1 ± 4.9	8.8 ± 1.4 ^a^	5.4 ± 1.5 ^a.b^	30.3 ± 5.6*	1912.8 ± 458.2*
	**Group2**	14.4 ± 3.1	7.0 ± 1.5 ^a^	3.8 ± 1.2 ^a.b^	25.2 ± 4.1	1368.6 ± 386.9	**CAP**	15.6 ± 3.0	8.1 ± 1.4 ^a^	5.1 ± 1.5 ^a.b^	28.8 ± 4.8*	1787.0 ± 614.0*

*TWL *total weight lifted, *CAP *Capsiate supplementation

*Note*: a= lower than Set-1; b= lower than Set-2. *= higher than Pre-intervention

For the acute resistance exercise session in leg press, there was a group × exercise (*F* = 3.779, *P =* 0.032, *η*^*2*^ *=* 0.17) and exercise x time interaction (*F* = 11.753, *P <* 0.001, *η*^*2*^ *=* 0.39) with a greater number of repetitions performed after 6 weeks of training compared to pre-training and a significant decrease across sets for both groups, however, there were no time × exercise × group interactions (*F* = 0.002, *P =* 0.998, *η*^*2*^ *=* 0.001). For the acute resistance exercise session in bench press, there was a greater number of repetition after 6 weeks of training compared to baseline (*F* = 19.737, *P* < 0.001, *η*^*2*^ *=* 0.52) and a significant decrease across sets for both groups (*F* = 185.601, *P* < 0.001, *η*^*2*^ *=* 0.91), but no time × set × group interaction were observed (*F* = 0.122, *P =* 0.886, *η*^*2*^ *=* 0.007).

Table 3 presents the changes on inflammatory response after 6 weeks of intervention in the Capsiate and placebo groups.

**Table 3:** Inflammatory response at baseline and after 6 weeks of chronic resistance training isolated and combined with Capsiate supplementation

Variables	Pre-intervention				After 6 weeks			
	**RT** (*n* = 11)		**RT + CAP** (*n* = 11)		**RT** (*n* = 9)		**RT + CAP** (*n* = 11)	
	**Rest**	**Post**	**Rest**	**Post**	**Rest**	**Post**	**Rest**	**Post**
**Log IL-6 (pg/mL)**	0.27 ± 0.11	0.32 ± 0.16	0.27 ± 0.11	0.32 ± 0.16	0.27 ± 0.08	0.24 ± 0.07	0.31 ± 0.23	0.25 ± 0.05
**Log IL-10 (pg/mL)**	0.48 ± 0.23	0.58 ± 0.45*	0.56 ± 0.27	0.75 ± 0.25*	0.58 ± 0.26	0.51 ± 0.30*	0.58 ± 0.22	0.68 ± 0.28*
**Log TNF-α (pg/mL)**	0.75 ± 0.15	0.91 ± 0.19^£^	0.95 ± 0.25	1.0 ± 0.28^£^	0.96 ± 0.34	0.85 ± 0.42	1.04 ± 0.34	1.04 ± 0.32
**Log sTNF-R (pg/mL)**	2.88 ± 0.24	2.93 ± 0.29	2.98 ± 0.25	3.12 ± 0.28	2.94 ± 0.24	3.03 ± 0.21	3.10 ± 0.25	3.25 ± 0.22

*Note*: *TNF-α *Tumor necrosis factor-α, *TNF-R *Soluble tumor necrosis factor-α receptor. £= exercise × time interaction with significant difference compared to Rest. *= a main effect of exercise

There was a significant exercise × time interaction for TNF-α (*F* = 4.926, *P =* 0.040, *η*^*2*^ *=* 0.22) with a significant increase post-exercise in relation to rest at pre-intervention (*P* = 0.009) but no time × set × group interaction (*F* = 2.190, *P =* 0.157, *η*^*2*^ *=* 0.11). For sTNF-r, there was a main effect of time (*F* = 13.449, *P =* 0.002, *η*^*2*^ *=* 0.46) and exercise (*F* = 28.545, *P <* 0.001, *η*^*2*^ *=* 0.64) but without time × set × group interaction (*F* = 0.123, *P =* 0.730, *η*^*2*^ *=* 0.008). There was a main effect of exercise for IL-10 (*F* = 4.964, *P =* 0.041, *η*^*2*^ *=* 0.24) with higher concentration post-exercise compared to rest, however, there was no significant time × set × group interaction (*F* = 0.197, *P =* 0.663, *η*^*2*^ *=* 0.012). There were not any significant main effects for IL-6 or interactions (*F* = 0.066, *P =* 0.801, *η*^*2*^ *=* 0.004).

## Discussion

We hypothesized chronic CAP supplementation would enhance the anti-inflammatory response to resistance training, improve muscular strength and enhance body composition outcomes. While CAP supplementation resulted in greater increases in lean body mass compared to placebo, there were no differences in the immune response to resistance exercise nor performance, with the exception of the bench press 1 RM test.

Based on previously reported acute increases in resistance exercise performance [6] with CAP supplementation, we hypothesized that if supplemented chronically and combined with resistance training, CAP would result in in greater volume accomplished per session, and this would translate to greater muscle hypertrophy. We reject this hypothesis. Although lean body mass increased in CAP, there were no differences between groups for training volume. The interaction between TRVP1 agonists and protein synthesis may explain this discrepancy. By agonizing the TRPV1 receptor, capsaicin increases sarcoplasmic reticulum calcium release and intracellular calcium concentrations and it been shown to increase mTORC1 phosphorylation and activation of downstream targets (p70S6K, S6, Erk1/2 and p38 MAPK) of protein synthesis [16]. In the absence of overload, capsaicin generated muscle hypertrophy in mice was associated with TRPV1-mediated activation of mTOR/p70S6K. Additionally, the same authors reported denervated soleus and gastrocnemius muscles atrophied by 32 and 49%, respectively, but CAP alleviated this atrophy [16]. Whether CAP supplementation augments resistance exercise induced increases in mTOR phosphorylation, and whether these increases in mTOR phosphorylation translate to chronic muscle hypertrophy requires further investigation.

We also hypothesized CAP would result in greater leg press and bench press 1RM and repetitions to failure volume following 6 weeks of training. We reject this hypothesis as both groups increased strength after 6 weeks of training with no differences between groups. Both groups also increased repetitions to failure following 6 weeks of training in the leg press and bench press. In afferent pain-conducting nerve fibers (nociceptors), chronic capsaicin administration and the resulting calcium influx results in a TRPV1 channel desensitization [17]. Long term *in vitro* administration of capsaicin has been shown to result in a large reduction and subsequent degradation of both internal and plasma membrane TRPV1 receptors [18]. Additionally, systemic administration of capsaicin to newborn mice resulted in a downregulation of TRPV1 receptors in neuronal but not vascular smooth muscle cells [17, 19]. If a similar down-regulation of TRPV1 receptors occurs in skeletal muscle, it may explain why acute CAP supplementation improved performance in Conrado de Freitas et al. [6], whereas chronic supplementation in the present study was ineffective.

In regards to the inflammatory response, TRPV1 activation by both heat (37–42 °C) and capsaicin have been shown to increase IL-6 mRNA expression in mouse myoblasts [4]. While the stress induced by exercise results in an anti-inflammatory response and the myokines involved can influence repair and regeneration response in skeletal muscle cells [20], the dose of CAP used in the present investigation did not potentiate the inflammatory response in untrained men. It is necessary to mentioned that the inflammatory response in our study was measured only immediately post-exercise and at the systemic level, therefore, more research is necessary to investigate cytokine kinetics (i.e. 30 min and 1 h after exercise) and also the paracrine and autocrine signaling with CAP supplementation.

A limitation to this study was our inability to sample blood or tissues. Therefore, more research is necessary to investigate the mechanism by which CAP could induce hypertrophy in humans, and to determine if chronic CAP ingestion decreases TRPV1 in skeletal muscle. The form and dose of capsaicin in the present study was well-tolerated and none of the subjects reported gastrointestinal distress.

## Conclusions

In summary, short term (6 weeks) chronic Capsiate supplementation in conjunction with resistance training increased fat-free mass and upper body strength but did not modulate the inflammatory response to exercise nor performance in young untrained men. The present study can be applied by coaches, trainers and sport nutritionist looking to improve fat-free mass gain and upper body strength during sub-acute chronic capsaicin analog supplementation combined with resistance training.

## Supplementary Information

**Additional file 1: Supplementary Table 1**. Resistance training program. **Supplementary Table 2**. Comparison between placebo and capsaicin on the dietary intake, macronutrient and micronutrients distribution.

## Data Availability

Data and publication materials can be provided upon request. Please contact corresponding author for this information.

## Abbreviations

CAP: Capsiate
TRVP-1: Transient Receptor Potential Vanilloid-1
mRNA: Messenger RNA
IL-6: Interleukin 6
TNF-Α: Tumor Necrosis Factor Alpha
IL-10: Interleukin 10
1RM: Maximum Repetition Test
TACO: Brazilian Food Composition Table
TBW: Total Body Water
ICW: Intracellular
ECW: Extracellular Water
FFM: Fat-Free Mas
FM: Fat Mass
FM%: Fat Mass
STNF-R: Soluble TNF-α Receptor
RMANOVA: Repeated Measures Analysis of Variance
SD: Standard Deviation
RPE: Rating of Perceived Exertion
