# Supplementary material for: Chronic capsiate supplementation increases fat-free mass and upper body strength but not the inflammatory response to resistance exercise in young untrained men: a randomized, placebo-controlled and double-blind study
Source: J Int Soc Sports Nutr. 2021 Jun 21;18:50. doi: 10.1186/s12970-021-00446-0 (PMC8218493; doi:10.1186/s12970-021-00446-0)
Supplement: Supplementary file 1 — Additional file 1: Supplementary Table 1. Resistance training program. Supplementary Table 2. Comparison between placebo and capsaicin on the dietary intake, macronutrient and micronutrients distribution. [file 12970_2021_446_MOESM1_ESM.docx]

**Supplementary Table 1**. Resistance training program.

|  | **Exercises** | **Sets** | **Repetition**  **1st to 3rd wks** | **Repetition**  **4th to 6th wks** |
| --- | --- | --- | --- | --- |
| **Day 1** | Bench Press | 3 | 10-12 | 8-10 |
|  | Seated Barbell Overhead | 3 | 10-12 | 8-10 |
|  | Lat Pull Down Machine | 3 | 10-12 | 8-10 |
|  | Triceps Pulley | 3 | 10-12 | 8-10 |
|  | Barbell Curl | 3 | 10-12 | 8-10 |
|  | Crunches | 3 | 15-20 | 20-25 |
|  | Lying Leg Raises | 3 | 15-20 | 20-25 |
|  |  |  |  |  |
| **Day 2** | 45º Leg Press | 3 | 10-12 | 8-10 |
|  | Back Squat | 3 | 10-12 | 8-10 |
|  | Leg Extensions | 3 | 10-12 | 8-10 |
|  | Lying Leg Curl | 3 | 10-12 | 8-10 |
|  | Calf Raises | 3 | 15-20 | 20-25 |
|  |  |  |  |  |
| **Day 3** | Incline Barbell Bench Press | 3 | 10-12 | 8-10 |
|  | Low Row Machine | 3 | 10-12 | 8-10 |
|  | Lying Barbell Triceps Extension | 3 | 10-12 | 8-10 |
|  | Scott Curls | 3 | 10-12 | 8-10 |
|  | Lateral Dumbbell Raises | 3 | 10-12 | 8-10 |
|  |  |  |  |  |
| **Day 4** | 45º Leg Press | 3 | 10-12 | 8-10 |
|  | Traveling Lunges | 3 | 10-12 | 8-10 |
|  | Leg Extensions | 3 | 10-12 | 8-10 |
|  | Lying Leg Curl | 3 | 10-12 | 8-10 |
|  | Seated Calf Raises | 3 | 15-20 | 20-25 |
|  | Crunches | 3 | 15-20 | 20-25 |
|  | Lying Leg Raises | 3 | 15-20 | 20-25 |

**Supplementary Table 2**. Comparison between placebo and capsaicin on the dietary intake, macronutrient and micronutrients distribution.

| **Dietary intake** | **Placebo (n=9)** | | **Capsaicin (n=11)** | |  |
| --- | --- | --- | --- | --- | --- |
| **Macronutrients** | **Pre** | **Post** | **Pre** | **Post** | **Group x Time** |
| CHO (g) | 312.8 ± 81.3 | 285.3 ± 56.8 | 306.1 ± 105.1 | 300.0 ± 59.1 | 0.630 |
| CHO (g/body weight) | 4.8 ± 1.9 | 4.3 ± 1.1 | 4.5 ± 1.6 | 4.4 ± 1.3 | 0.460 |
| PRO (g) | 107.1 ± 33.1 | 116.2 ± 21.4 | 96.0 ± 26.6 | 123.6 ± 24.7 | 0.320 |
| PRO (g/body weight) | 1.6 ± 0.6 | 1.7 ± 0.4 | 1.4 ± 0.4 | 1.8 ± 0.5 | 0.212 |
| LIP (g) | 76.0 ± 21.4 | 86.0 ± 18.5 | 73.7 ± 17.7 | 85.4 ± 13.2 | 0.862 |
| LIP (g/body weight) | 1.1 ± 0.4 | 1.3 ± 0.4 | 1.1 ± 0.3 | 1.3 ± 0.3 | 0.867 |
| Total Intake (kcal) | 2361.3± 524.8 | 2430.8 ± 369.7 | 2269.8± 623.4 | 2462.4± 317.1 | 0.674 |
| Total Intake (kcal/body weight) | 36.0 ± 12.4 | 36.5 ± 7.8 | 33.4 ± 9.9 | 36.5 ± 8.4 | 0.512 |
| **Micronutrients** |  |  |  |  |  |
| Ca (mg) | 547.5 ± 129,2 | 474.0 ± 223.9 | 492.9 ± 163.2 | 621.23 ± 283.9 | 0.116 |
| Ca (mg/body weight) | 8.4 ± 4.1 | 7.5 ± 4.6 | 7.2 ± 2.4 | 9.0 ± 3.5 | 0.100 |
| Mg (mg) | 230.1 ± 80.2 | 252.2 ± 44.9 | 214.3 ± 57.7 | 262.1 ± 39.7 | 0.444 |
| Mg (mg/body weight) | 3.6 ± 1.7 | 3.8 ± 1.0 | 3.1 ± 0.7 | 3.9 ± 0.9 | 0.275 |
| Zn (mg) | 10.7 ± 3.5 | 9.8 ± 2.4 | 9.6 ± 2.8 | 10.4 ± 2.8 | 0.401 |
| Zn (mg/body weight) | 0.2 ± 0.1 | 0.1 ± 0.0 | 0.1 ± 0.0 | 0.1 ± 0.0 | 0.457 |
| Iron (mg) | 15.5 ± 4.7 | 15.2 ± 2.9 | 15.8 ± 3.6 | 16.8 ± 3.7 | 0.611 |
| Iron (mg/body weight) | 0.2 ± 0.1 | 0.2 ± 0.1 | 0.2 ± 0.1 | 0.2 ± 0.1 | 0.528 |
| Se (mg) | 109.5 ± 30.1 | 119.9 ± 14.3 | 97.5 ± 25.2 | 110.2 ± 25.1 | 0.893 |
| Se (mg/body weight) | 1.7 ± 0.6 | 1.8 ± 0.3 | 1.4 ± 0.4 | 1.6 ± 0.4 | 0.828 |
| Vit A (mg) | 654.6 ± 430.1 | 468.1 ± 235.9 | 578.2 ± 404.1 | 565.1 ± 221.3 | 0.417 |
| Vit A (mg/body weight) | 10.0 ± 6.9 | 7.3 ± 4.4 | 8.4 ± 6.0 | 8.4 ± 3.7 | 0.389 |
| Vit C (mg) | 36.2 ± 18.3 | 30.9 ± 13.9 | 32.1 ± 10.4 | 35.6 ± 15.1 | 0.315 |
| Vit C (mg/body weight) | 0.6 ± 0.3 | 0.5 ± 0.3 | 0.5 ± 0.2 | 0.5 ± 0.2 | 0.346 |
| Vit D (mg) | 3.3 ± 1.9 | 3.3 ± 1.5 | 2.4 ± 1.1 | 2.5 ± 1.0 | 0.934 |
| Vit D (mg/body weight) | 0.05 ± 0.03 | 0.05 ± 0.03 | 0.04 ± 0.01 | 0.04 ± 0.01 | 0.985 |
| Vit E (mg) | 12.4 ± 3.6 | 19.1 ± 4.5 | 10.1 ± 4.5 | 16.6 ± 3.6 | 0.938 |
| Vit E (mg/body weight) | 0.2 ± 0.1 | 0.3 ± 0.1 | 0.2 ± 0.1 | 0.3 ± 0.1 | 0.909 |

**Note:** CHO= carbohydrate; PRO= protein; LIP= lipids; Ca=Calcium (mg); Mg= magnesium (mg), Zn= Zinc (mg); Se= Selenium (mg); Vit= Vitamins (mg).
